# Machine Learning and Medication Adherence: Scoping Review

**DOI:** 10.2196/26993

**Published:** 2021-11-24

**Authors:** Aaron Bohlmann, Javed Mostafa, Manish Kumar

**Affiliations:** 1 Carolina Population Center University of North Carolina at Chapel Hill Chapel Hill, NC United States; 2 Public Health Leadership Program University of North Carolina at Chapel Hill Chapel Hill, NC United States

**Keywords:** machine learning, medication adherence, adherence monitoring, adherence prediction, medication compliance, health technology

## Abstract

**Background:**

This is the first scoping review to focus broadly on the topics of machine learning and medication adherence.

**Objective:**

This review aims to categorize, summarize, and analyze literature focused on using machine learning for actions related to medication adherence.

**Methods:**

PubMed, Scopus, ACM Digital Library, IEEE, and Web of Science were searched to find works that meet the inclusion criteria. After full-text review, 43 works were included in the final analysis. Information of interest was systematically charted before inclusion in the final draft. Studies were placed into natural categories for additional analysis dependent upon the combination of actions related to medication adherence. The protocol for this scoping review was created using the PRISMA-ScR (Preferred Reporting Items for Systematic Reviews and Meta-Analyses Extension for Scoping Reviews) guidelines.

**Results:**

Publications focused on predicting medication adherence have uncovered 20 strong predictors that were significant in two or more studies. A total of 13 studies that predicted medication adherence used either self-reported questionnaires or pharmacy claims data to determine medication adherence status. In addition, 13 studies that predicted medication adherence did so using either logistic regression, artificial neural networks, random forest, or support vector machines. Of the 15 studies that predicted medication adherence, 6 reported predictor accuracy, the lowest of which was 77.6%. Of 13 monitoring systems, 12 determined medication administration using medication container sensors or sensors in consumer electronics, like smartwatches or smartphones. A total of 11 monitoring systems used logistic regression, artificial neural networks, support vector machines, or random forest algorithms to determine medication administration. The 4 systems that monitored inhaler administration reported a classification accuracy of 93.75% or higher. The 2 systems that monitored medication status in patients with Parkinson disease reported a classification accuracy of 78% or higher. A total of 3 studies monitored medication administration using only smartwatch sensors and reported a classification accuracy of 78.6% or higher. Two systems that provided context-aware medication reminders helped patients to achieve an adherence level of 92% or higher. Two conversational artificial intelligence reminder systems significantly improved adherence rates when compared against traditional reminder systems.

**Conclusions:**

Creation of systems that accurately predict medication adherence across multiple data sets may be possible due to predictors remaining strong across multiple studies. Higher quality measures of adherence should be adopted when possible so that prediction algorithms are based on accurate information. Currently, medication adherence can be predicted with a good level of accuracy, potentially allowing for the development of interventions aimed at preventing nonadherence. Monitoring systems that track inhaler use currently classify inhaler-related actions with an excellent level of accuracy, allowing for tracking of adherence and potentially proper inhaler technique. Systems that monitor medication states in patients with Parkinson disease can currently achieve a good level of classification accuracy and have the potential to inform medication therapy changes in the future. Medication administration monitoring systems that only use motion sensors in smartwatches can currently achieve a good level of classification accuracy but only when differentiating between a small number of possible activities. Context-aware reminder systems can help patients achieve high levels of medication adherence but are also intrusive, which may not be acceptable to users. Conversational artificial intelligence reminder systems can significantly improve adherence.

## Introduction

Health care costs will continue to rise into the foreseeable future unless technology is implemented that substantially increases the efficiency of care delivery. Machine learning is a technology with the potential to automate many health care processes, including actions that impact medication adherence. Medication adherence is an important issue because approximately 50% of patients with chronic disease are not adherent to their medications, thus increasing medical costs and avoidable human suffering [1].

Recently, reviews were published that discuss the effectiveness of using machine learning to improve medication adherence. Awan et al [2] investigated the use of machine learning to improve the care of patients with heart failure. Two of the studies mentioned in this review used machine learning to predict medication adherence. Another review, written by Cresswell et al [3], investigated the use of data-driven artificial intelligence (AI) in computerized systems that support health and social care. Of the articles mentioned, 2 of them focused on using machine learning to improve medication adherence. One used neural networks and computer vision to enhance outpatient adherence to anticoagulant medications. The other adherence study discussed using an AI system to provide personalized support to patients taking warfarin.

In contrast to previous works, this review is focused more generally on the use of machine learning within the confines of medication adherence. By using a broader perspective, this paper provides high-level insight into this area of study, which is not possible with more narrowly focused examinations. Medication adherence is a complex problem that can be engaged from multiple angles using machine learning. This paper serves as a way to quickly learn about different approaches, their current level of development, and obstacles that need to be overcome to use machine learning more effectively toward improving medication adherence.

This review also categorizes and summarizes how machine learning has been used to execute actions related to medication adherence in academic literature. Within each category, common themes will be explored to find gaps and opportunities for future work.

## Methods

### Eligibility Criteria

The eligibility criteria were developed through collaboration between authors AB and JM. Papers included in this review carried out at least one action related to machine learning and medication adherence. Studies also had to test out their application of machine learning to medication adherence using either real patients, research participants, or simulations. The ABC taxonomy was used to define medication adherence for this study. This taxonomy defines medication adherence as “the process by which patients take their medications as prescribed, composed of initiation, implementation and discontinuation” [4].

### Information Sources

The following databases were searched to identify relevant papers: PubMed, Scopus, IEEE, ACM Digital Library, and Web of Science. These databases were selected by the Laboratory of Applied Informatics Research (LAIR) team as representing the largest and most extensive coverage of studies that investigate applying machine learning to medication adherence.

### Search Query

Search queries for this paper were created with the help of Rebecca Carlson and Fei Yu, both of whom are experienced research librarians. Search terms were selected by reviewing exhaustive term lists provided by the research librarians on the topics of machine learning and medication adherence. The same keywords were selected for all five databases by combining the lists of machine learning and medication adherence terms using an AND operator. The final search was conducted on April 30, 2020. When searching PubMed, Medical Subject Headings (MeSH) terms were incorporated to provide broad coverage of related vocabulary. Following PubMed, this same query strategy was used to search Scopus but with Emtree Terms in place of the MeSH terms. For IEEE, ACM Digital Library, and Web of Science, the keywords were used exclusively to discover relevant papers. Examples of the search queries are provided in section A of Multimedia Appendix 1.

### Selection of Evidence Sources

The search queries found 504 studies that contain both machine learning and medication adherence terms. After removing duplicate studies, 417 papers were identified for review. The title and abstract review were conducted using a two-person team consisting of authors AB and MK. The title and abstract evaluation reduced the total number of relevant papers to 54. Next, a sample of 20 articles was screened. This initial screening was done to ensure that information was collected in a consistent manner using a data charting template. This template was developed through collaboration with the LAIR team and the primary author of this work. After finalization of the data charting template, all 43 papers were reviewed in full by AB and MK. Studies that passed the full-text review focused on both medication adherence and machine learning. A focus on medication adherence and machine learning was essential to reduce the number of studies to a manageable list of highly relevant works. Included works also evaluated the use of machine learning for medication adherence actions using either patients, research participants, or simulations. This testing requirement was used to exclude studies that are still in the early development stages and to make sure that included works contained all of the relevant data charting elements. Next, the results were grouped into natural categories and analyzed according to the combination of medication adherence actions in each study. This grouping of related studies was carried out by AB, JM, and MK.

**Figure 1:**
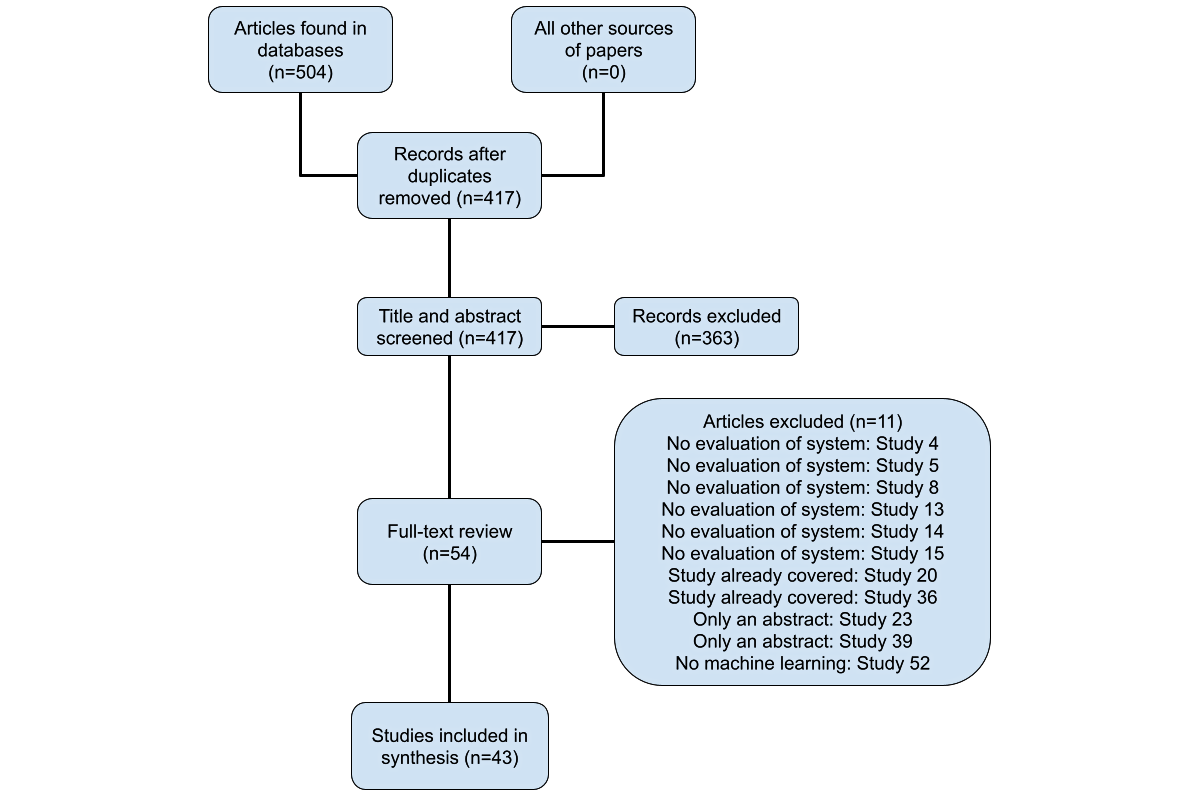
Article review process.

Figure 1 shows the article review process and provides reasons why specific studies were excluded during the full-text review.

### Data Charting Process

A data charting form was created and updated throughout the review process as needed. This form was used to guide the selection of relevant information throughout the review process and was tested to ensure consistency across different articles during the sample screening. The items included in the data charting template are listed in the next section.

### Data Items

Data charted for this review includes the article title, publication date, study number, study goals, main study results, disease states, predictors of medication adherence, types of machine learning used, number of participants, data collection methods, actions related to medication adherence, adherence measurements, limitations, and inclusion or exclusion status.

### Categorization of Studies

Following data charting, actions related to medication adherence were determined for each study. Next, articles were grouped for further analysis according to the combinations of medication adherence actions included in each work. AB, JM, and MK conducted the categorization process, and disagreements were discussed until a consensus was reached. Following categorization, the data charting documents for these works were reviewed to find relevant themes. A general analysis was also conducted before categorization in terms of the number of publications per year, articles per disease state, and publications per database type.

## Results

### Results of Individual Sources of Evidence

This paper includes the analysis of 43 studies selected for inclusion. The main results of this review are listed in Tables 1-3. More detailed data charting tables are also available in section B of Multimedia Appendix 1.

**Table 1:** Summary of studies that predict medication adherence.

Study	Year	Data collection	Algorithms	Disease states	Strong predictors of adherence^a^	Adherence metric predicted	Main outcome metric^b^
Walczak and Okuboyejo [5]	2017	Self-reported questionnaire	ANNs^c^	General	Variable strength not discussed in detail	Self-reported reasons for medication adherence status	79.3% accuracy when predicting reasons for poor adherence
Son et al [6]	2010	Self-reported questionnaire	SVM^d^	Heart failure	Disease severity classification, medication knowledge, gender, daily medication frequency, marital status	Self-reported medication adherence status	77.6% accuracy when predicting medication adherence
Aziz et al [7]	2020	Self-reported questionnaire	ANNs, RF^e^, SVM	Hypertension	Education level, marital status, general overuse, monthly income, specific concern	Self-reported medication adherence status	79% accuracy when predicting medication adherence
Aznar-Lou et al [8]	2018	Pharmacy claims data	LR^f^	General	Medication cost for low-income individuals	Filling a medication within 2 months of issuance	Statistically significant association between medication cost and medication adherence in low-income group
Zhang and Meltzer [9]	2016	Self-reported questionnaire	LR	General	Time between social security check arrival and filling prescriptions, age, specific chronic conditions	Self-reported adherence status	Statistically significant association between medication adherence and time difference between receiving social security check and filling drugs
Aznar-Lou et al [10]	2017	Pharmacy claims data	LR	High-cost diseases	Age, nationality, number of chronic conditions, active disease, being treated by primary care provider	Patient filling their medication within 1 month of issuance	Statistically significant association between medication adherence and age, nationality, number of chronic conditions, specific active diseases, type of care provider
Haas et al [11]	2019	Self-reported questionnaire	RF	Fibromyalgia	Type of medication, years of treatment, dosage, age, gender, region of residence	Self-reported adherence status on a health forum	67.8% accuracy when predicting medication adherence
Lu et al [12]	2005	Self-reported questionnaire	ANNs, SVM	HIV	Viral load, drug abuse, alcohol abuse, psychiatric diagnosis, missed clinic visits, housing, HIV-related inpatient medical care	Self-reported adherence class ranging from class one to four	100% accuracy when predicting adherence class but sample size of only 33
Franklin et al [13]	2016	Pharmacy claims data	LR	High cholesterol	Age, sex, race, specific statin medication, count of health services score, cardiovascular disease status, cardiovascular procedure status, comorbidities, initial statin fill behavior	Proportion of days covered of 0.8 or higher	84.2% accuracy when predicting medication adherence
Karanasiou et al [14]	2016	Clinical estimation	ANNs, SVM, RF, J48, random tree, logistic tree, classification tree, rotation forest, radial basis function, Bayesian network, Naive Bayes	Heart failure	Specific medical conditions, specific medications, medication dose, medication frequency	Provider determined adherence status to medications and lifestyle changes	91% sensitivity when predicting medication adherence
Bourdès et al [15]	2011	Self-reported questionnaire	ANNs, LR	ACS^g^	Coronary artery bypass graft status, overweight with BMI between 25 and 30, hypercholesterolemia, education level	Self-reported concurrent use of an ace inhibitor or angiotensin receptor blocker, beta blocker, statin, and a blood thinning agent	0.80 AUC^h^ when predicting medication adherence
Kim et al [16]	2019	Self-reported questionnaire	Classification tree	Smoking cessation	Belief in medication safety, taste and sensory properties, exposure to others smoking, quitting confidence	Self-reported adherence during a phone interview	2.22 importance score for belief in medication safety, 1.84 importance score for taste and sensory properties, 1.67 importance score for exposure to other smokers, 1.15 importance score for quitting confidence
Kardas et al [17]	2020	Pharmacy claims data	LR	Chronic disease	Age, medication cost, medication class	Not picking up a medication within 1 month of issuance	Statistically significant association found between age and medication adherence
Desai et al [18]	2019	Pharmacy claims data	LR	Fibromyalgia	Gender, age, race, comorbidity score, medication type, health coverage, emergency room visits	Medication possession ratio of 80% or higher	AUC of 0.6224 when predicting medication adherence
Elahinia et al [19]	2017	Discharge summaries	Natural language processing	General	Keywords frequently found in the discharge summaries of nonadherent patients	Adherence status based on professional interpretation of discharge summaries	Statistically significant association between category 1, 2, and 3 keywords with medication adherence

^a^Predictor strength was based on individual study results.

^b^Main outcome metric based on judgement of the research team after careful consideration of all results presented in the individual study.

^c^ANN: artificial neural network.

^d^SVM: support vector machine.

^e^RF: random forest.

^f^LR: logistic regression.

^g^ACS: acute coronary syndrome.

^h^AUC: area under the curve.

**Table 2:** Summary of studies that monitor medication adherence.

Study	Year	Algorithms	Sensors	Diseases	Data analyzed using machine learning to determine adherence	Main outcome metric^a^
Hezarjaribi et al [20]	2016	Classification tree	Body worn	Chronic disease	Smartwatch movement data collected during one of five tasks: drinking water, taking a pill while sitting, taking a pill while standing, writing, eating	78.6% detection accuracy using only smartwatch sensors
Fozoonmayeh et al [21]	2020	RF^b^, LR^c^, SVM^d^, boosted tree	Body worn	General	Smartwatch movement data collected during medication ingestion or other predetermined tasks	98.3% detection accuracy using smartwatch sensors
Aldeer et al [22]	2019	RF, SVM	Smart pill bottle	General	Medication bottle movement and cap sensor data collected during medication ingestion or other predetermined tasks	90% accuracy when using all smart bottle sensors
Ntalianis et al [23]	2019	ANNs^e^	Audio recording device	Asthma/COPD^f^	Sound recordings of inhaler use broken down into different inhaler use actions like breathing in, actuation, and breathing out	95.86% classification accuracy using audio files
Tucker et al [24]	2015	NB^g^, IBk^h^, SVM, J48, RF	Kinect	Parkinson disease	3D movement scans of patients with Parkinson disease in different adherence states	97% classification accuracy when determining medication status for a single patient, 78% classification accuracy when determining medication status of multiple patients
Ma et al [25]	2018	RF	Body worn	General	Movement data collected using a smartwatch during six predefined activities: medication intake with nondominant hand, medication intake with dominant hand, walking, texting, writing with a pen, drinking water	Recall of 1.00 with a precision of 0.80 for medication intake classification using smartwatch sensors
Nousias et al [26]	2016	SVM, RF, AdaBoost	Audio recording device	Asthma/COPD	Audio recordings of inhaler use, breathing in, breathing out, and noise not related to medication use	97.59% classification accuracy using audio recordings
Bilodeau and Ammouri [27]	2011	Petri net	Camera	General	Video footage of people taking medications in which the head, hands, and medication bottle are clearly visible	Correctly identified medication taking 9 out of 12 times
Aldeer et al [28]	2019	SVM, RF	Smart pill bottle	General	Patient-specific movement profile data collected using pill bottle movement and cap sensors	91% accuracy identifying the movement patterns of a specific patient
Zhang et al [29]	2019	ANNs	Body worn	Parkinson disease	Movement data of patients with Parkinson disease collected using a smartphone	83.4% classification accuracy using body worn sensors
Pettas et al [30]	2019	ANNs, RF	Audio recording device	Asthma/COPD	Audio recordings of sounds related to inhaler use or recordings of non-inhaler–related sounds	Classification accuracy of 93.75% using audio recordings
Moldovan et al [31]	2018	ANNs, LR, RF, decision tree	Body worn	Dementia	Movement data of patients with dementia during medication ingestion and other predetermined tasks collected using four body worn motion sensors	Classification precision of 0.91 using body worn sensors
Kikidis et al [32]	2015	ANNs	Audio recording device	Asthma/COPD	Audio recordings that contain either an inhaler actuation or a non-inhaler–related sound	99.5% classification accuracy using audio recordings

^a^Main outcome metric based on judgement of the research team after careful consideration of all results presented in the individual study.

^b^RF: random forest.

^c^LR: logistic regression.

^d^SVM: support vector machine.

^e^ANN: artificial neural network.

^f^COPD: chronic obstructive pulmonary disease.

^g^NB: Naive Bayes.

^h^IBk: instance-based classifier with parameter k.

**Table 3:** Summary of studies that monitor and attempt to improve medication adherence.

Study	Year	Sensors	Adherence intervention	Disease states	Algorithms	Data analyzed using machine learning to determine adherence	Main outcome metric^a^
da Silva et al [33]	2019	Smart medication cabinet, cameras, movement sensors, light sensors, thermostat, smart TV sensors, smartphone sensors, smartwatch sensors, door sensors, blood pressure sensors	Context-aware medication reminder prompts	Hypertension	C4.5, random tree, RepTree	Medication ingestion confirmed by medication cabinet using door sensors, RFID^b^ tags, video recording, patient daily patterns monitored using motion sensors in house, light sensors, thermometer sensors, smart TV sensors, smartphone sensors, home door sensors, blood pressure sensors	95.10% medication adherence level for people using the system
Lundell et al [34]	2007	Smart medication cabinet, motion sensors, refrigerator sensors, smartphone sensors, smartwatch sensors, bed sensors, front door sensors	Context-aware medication reminder prompts	General	Dynamic Bayesian network	Medication ingestion confirmed using pill tray lid sensors, patient daily patterns tracked using motion sensors in house, refrigerator sensors, smartphone sensors, smartwatch sensors, bed sensors, front door sensors	92% medication adherence for people using the system
Silva et al [35]	2018	Smart medication cabinet, computer sensors, tablets sensors, TV sensors, smartwatch sensors, smartphone sensors	Context-aware medication reminder prompts	Hypertension	J48, RF^c^, RepTree, random tree	Medication ingestion determined by smart drug cabinet that uses RFID tags and door sensors, patient daily patterns tracked using computer sensors, tablet sensors, smart TV sensors, smartphone sensors, smartwatch sensors	95.2% classification accuracy using all available sensors
Silva et al [36]	2016	Smart medication cabinet, motion sensors, lighting sensors, cameras, smartphone sensors, TV sensors	Context-aware medication reminder prompts	General	J48, RepTree, random tree	Medication ingestion determined using smart medication cabinet with RFID tags and camera system, patient daily activity tracked using motion sensors in home, light sensors, surveillance cameras, smartphone sensors, smart TV sensors	Testing of a prototype system was conducted but no statistical results were presented
Nousias et al [37]	2018	Microphone	Visualization of inhaler use process	Asthma, COPD^d^	GMM^e^, SVM^f^, RF, AdaBoost	Audio recordings of inhalation, exhalation, inhaler actuation, and background noise	98% classification accuracy using audio recordings
Persell et al [38]	2020	None	Conversational AI^g^ adherence coaching	Hypertension	Not discussed in detail	Blood pressure, weight, self-reported adherence, number of medications, number of dose increases or substitutions, compliance with: diet, exercise, sleep duration	No significant differences in adherence when comparing patients using smartphone coaching app vs those not using the app
Brar Prayaga et al [39]	2018	None	Conversational AI refill reminder system	Chronic disease	Not discussed in detail	Medication names, gender, number of refills processed using the system, patient responses using keypad or unstructured verbal responses	Text messaging reminder system improved adherence significantly with 14.07% more refills than the control group receiving traditional reminders
Chaix et al [40]	2019	None	Conversational AI medication reminder system	Breast cancer	Not discussed in detail	Patient verbal responses to medication reminder: “yes I took it,” “no i didn’t take it,” “send me a message in 15 minutes”	Average compliance improved significantly in the chatbot group with 20% higher adherence levels when compared to the control group
Curci et al [41]	2017	Smart pill bottle	Medication reminder, nonadherence messaging to provider	General	RF, RIPPER, Bayesian networks, SVM, ANNs^h^	Movement sensor data recorded by pill bottle, patient response via cell phone app answering if they took the medication when medication ingestion is suggested by movement data, patient response to scheduled reminders indicating if the medication was taken or not	90% classification accuracy using medication bottle movement sensors
Labovitz et al [42]	2017	Camera	Medication reminder, adherence history visualization	Ischemic stroke	Not discussed in detail	Video recordings of medication ingestion using a smartphone and pill counts for patients in group one, blood concentration of medication and pill counts for patients in group two	Patient adherence using the AI platform was 90.5% compared with 100% using blood samples to measure drug levels

^a^Main outcome metric based on judgement of the research team after careful consideration of all results presented in the individual study.

^b^RFID: radio-frequency identification.

^c^RF: random forest.

^d^COPD: chronic obstructive pulmonary disease.

^e^GMM: Gaussian mixture model.

^f^SVM: support vector machine.

^g^AI: artificial intelligence.

^h^ANN: artificial neural network.

### Synthesis of Results

Before dividing the studies into categories, they were examined as a whole to determine the distribution of articles concerning time, disease states, and database type.

Table 4 displays the number of publications per year that apply machine learning to medication adherence. For the past few years, this topic has generated a growing number of publications, indicating that there is interest building around this topic.

Table 5 illustrates the distribution of different disease states within the included articles. The largest group in this graph is general and consists of 12 studies. General indicates that these studies did not focus on any specific disease or group of diseases. The remaining 32 papers, except a single study focused on high-cost medications, looked at chronic diseases.

**Table 4:** Number of included studies per year.

Year of publication	Studies included, n
2005	1
2007	1
2010	1
2011	2
2013	1
2015	2
2016	6
2017	7
2018	6
2019	12
2020	4

**Table 5:** Number of included studies per disease group.

Disease groups	Studies included, n
Nonspecific	12
Cardiovascular diseases	10
Pulmonary diseases	5
General chronic diseases	4
Diseases of aging	3
Psychiatric diseases	2
Infectious diseases	2
Chronic pain	2
Smoking cessation	1
Cancer	1
Diseases with expensive medications	1

### Analysis of Natural Categories

Following the general analysis, actions related to medication adherence were determined. The three identified actions were prediction of adherence, adherence monitoring, and adherence interventions. Next, studies were grouped into natural categories for further analysis according to the combination of medication adherence actions that they contain. The following natural groups were identified: prediction of adherence only, monitoring of adherence only, monitoring with an intervention to improve adherence, all three medication adherence actions simultaneously, and prediction with monitoring.

### Studies That Predict, Monitor, and Intervene to Improve Medication Adherence

The fourth group contained 3 articles that predicted adherence, monitored adherence, and intervened to improve medication adherence [43-45].

The first of these studies allowed patients to request medication refills using a conversational AI SMS text messaging system [43]. The system would prompt the user when they needed to request a refill for their medication. The predictors used within this study were age, gender, spoken language, address (used to determine social determinants of health), race, ethnicity, and patient response via either structured response or using free-text entry. This study used an artificial neural network to predict if the patient would use this system to request a medication refill. Machine learning was also used for conversational AI, but the specifics were not discussed within the article. This system was not restricted to any specific disease state and was tested using approximately 99,000 patients. The limitations are that data was only collected over 3 months and that 8% of free-text responses were not correctly understood by the conversational AI.

The next work used data from the 99DOTS (Directly Observed Treatment Short Course) program, which monitors medication adherence of patients with tuberculosis [44]. Patients in this program used a phone to enter a random code for each dose of the medication they took. Other data collected for 99DOTS included: demographic data (age, weight, gender, treatment center ID), treatment start date, treatment end date, adherence string, and treatment outcome if applicable. This data was manipulated to create both static and time series predictors. Random forest, linear regression, and support vector machine (SVM) were applied to the static predictors to determine daily nonadherence risk, treatment success, and how to best allocate limited resources. A deep network called LEAP, which is capable of using time series predictors, was also used to predict these same outcomes. LEAP had the best prediction accuracy of the machine learning methods used. This system was tested using the monitoring data of about 17,000 people.

The next paper used face recognition software and computer vision to monitor medication adherence of 53 patients that had schizophrenia [45]. Patients in this study used a smartphone camera to record medication ingestion and submitted those recordings to the research team. Face recognition and computer vision techniques were then used to flag patients engaging in suspicious behavior, which indicated that long-term adherence was unlikely. This system also reminded patients to take their medications at a specific time each day. The main limitation was that patients were allowed to choose either direct in-person observation or monitoring via the app thus introducing bias into the study.

### Studies That Predict and Monitor Medication Adherence

The next group had 2 articles that predicted and monitored medication adherence but did not introduce any medication adherence intervention [46,47].

The first of these articles used data collected during hospital stays to generate predictors. Of these predictors, disease severity and biomarkers (breath, saliva, blood) had the largest impact on the model’s accuracy [46]. The overall goals of this study were to predict the adherence risk of a given patient with heart failure and to monitor their ongoing adherence. Nine different classifiers were tested for this study, including random forest, logistic model trees, J48, simple classification/regression tree-CART, rotation forest, radial basis function network, SVM, Bayesian network, and Naive Bayes. Of all of these methods, random forest performed the best in terms of classification accuracy. The data of 29 patients was used to create this system.

The second paper used interactive voice response assessments to predict future adherence and to monitor current medication adherence in patients with depression [47]. The data used for this study was collected using interactive voice response assessments of 208 patients. These assessments were then used to create predictors. Of these predictors, the following added to the power of the model: past medication adherence, age, and physical functioning at baseline. Only logistic regression was used to predict future medication adherence.

## Discussion

### General Discussion

This is the first review to focus broadly on applying machine learning to medication adherence. This study provides a general summary of the topic and categorizes literature according to the combination of medication adherence actions. Within each category, common themes were explored and opportunities for future work were identified.

The application of machine learning to medication adherence is a topic still in its infancy that has become more prevalent over the last few years. This technology is typically being applied to patients with chronic diseases that require long-term medication use. In fact, 29 of the 43 studies looked at using machine learning to impact medication adherence within the context of chronic disease [6,7,11-15,17,18,20,23,24,26, 29-33,35,37-40,42-47]. This is not surprising, since patients with chronic diseases often have a high medication burden and poor medication adherence over an extended period of time.

### Discussion of Studies That Predicted Medication Adherence

Twenty predictors of medication adherence were found to be important across two or more independent studies [6-19]. These predictors included: education level, marital status, income, gender, geographic location, emergency care interventions, age, race, ethnicity, disease severity, comorbidities, medication cost, insurance coverage, substance abuse, medication beliefs, medication knowledge, medication dose, medication frequency, initial medication adherence, and current medications. This suggests that it may be possible to build models that maintain a high level of accuracy even when applied to a different set of patients. In fact, 1 study in this review applied an established algorithm developed using the MEPS data set to a new data set pulled from a social health forum. This study managed to achieve a modest prediction accuracy of 67.8%, despite being trained on a completely different data set. However, this required careful manipulation of the input data to ensure it was formatted appropriately for the established prediction algorithm. In addition, two predictor variables were discarded to improve prediction accuracy. One limitation of several studies that predicted medication adherence is that they did not take socioeconomic factors into consideration [16,18]. Socioeconomic factors were determined to be strong predictors of adherence by multiple studies within this review and should be included when possible [7-9,17].

Similar methods of data collection were also used in works that solely predicted medication adherence. The most popular method of data collection and determining adherence was self-reported questionnaires. In fact, 8 of the 15 studies in this group determined medication adherence using self-reported questionnaires [5-7,9,11,12,15,16]. Although collecting data with questionnaires is attractive due to its simplicity and low cost, it typically overestimates adherence when compared to other data collection methods. When a questionnaire is used, a validated self-adherence measure is preferred, and efforts should be made to reduce negative social pressures associated with reporting nonadherence [48]. Pharmacy claims data was also used by one-third of these studies for both patient information and to determine adherence [8,10,13,17,18]. Pharmacy claims data is generally considered more reliable than self-reported questionnaires but still may overestimate adherence since it does not actively investigate whether the patient is taking the medication or not.

Additionally, studies that only predicted medication adherence used many of the same algorithms. Of 15 studies, 13 in this group used either logistic regression, artificial neural network, SVM, or random forest algorithms. Some of these works compared the different types of algorithms to determine which was the most accurate [7,12,14,15]. However, more publications need to be generated before any type of consensus can be reached on this issue.

Furthermore, 13 of the 15 studies that attempted to predict medication adherence used either a self-reported adherence metric or medication filling data to train their models [5-13,15-18]. This adherence metric along with predictor variables was provided during the training of each algorithm. Following model training, the adherence metric was not provided to the model and instead was predicted based only on the predictor variables. Of the 6 studies that provided prediction accuracy, 5 of them reported accuracy of 77.6% or higher [5-7,12,13]. This level of accuracy is impressive, and this data could potentially be leveraged to prevent medication adherence issues before they occur. However, the specificity of the algorithm in question would need to be carefully investigated to ensure that it is not overpredicting poor medication adherence, potentially wasting resources directed at preventative measures. Within this group, 5 other studies reported a statistically significant association between predictor variables and medication adherence as their main result [8-10,17,19]. One study found that both nationality and provider type are significant predictors of medication adherence [10]. These two predictors should be investigated in follow-up studies to determine if they remain significant across multiple investigations. Another study within this review found a significant association between specific keywords and medication adherence, which can be used to predict medication adherence based on free-text discharge summaries [19]. More studies should be conducted to find keywords associated with medication adherence since a large amount of medical documentation is stored as free text. This is especially useful in analyzing data from less sophisticated clinical systems with less structured documentation.

### Discussion of Studies That Monitored Medication Adherence

Machine learning was also used to monitor medication adherence. The main purpose of all 13 studies within this group was to develop new ways of monitoring medication adherence with the aid of sensors and machine learning. One popular approach, used in 6 of these 13 studies, was to attach sensors to the container holding the patient’s medication [22,23,26,28,30,32]. Four of these studies attached a microphone to an inhaler, and the other 2 used a smart pill bottle to track medication adherence. Direct attachment of sensors to medication holding devices is an established method of tracking medication adherence since the opening or activation of the medication holding device can be directly recorded. However, this form of data collection still has some major drawbacks outside of smaller deployments because it requires copious hardware to scale. This hardware would also likely be disposable unless additional effort was required on the part of the patient to load the medication or medication container into a reusable device.

Another popular approach was to use portable smart devices to track medication adherence. Of these studies, 6 used portable sensors found in common consumer electronics like smartwatches or smartphones [20,21,25,27,29,31]. One of these studies required users to actively record ingestion using a smartphone camera, and the other 5 recorded movement data using smartwatch sensors. Having patients track their medication ingestion with a camera every time they take a dose is accurate since the act of ingestion is being directly observed. However, it is unclear if patients would actually use the system and might find this level of observation overly intrusive.

Studies that only monitored medication adherence used many of the same machine learning algorithms. Of the 13 studies, 11 in this group used logistic regression, artificial neural network, SVM, or random forest algorithms [21-26,28-32]. Several studies compared the accuracy of these different algorithms, but this was not enough to generate any meaningful conclusions [22,26,28,30].

Moreover, works that only monitored medication adherence often built systems that classified user activities based on data provided by sensors. Several studies attempted to monitor the use of inhalers by analyzing audio recordings of inhaler use [23,26,30,31]. All 4 of these studies were able to classify sounds of inhaler use with an accuracy of 93.75% or higher. This is especially impressive when one considers that 3 of the studies also introduced background noise samples to make the evaluation more comparable to real-world environments [26,30,31]. All four systems also classified different parts of inhaler use such as inhalation, actuation of the inhaler, and exhalation. This allows these systems to track both medication adherence and proper inhaler technique simultaneously. Patients using a device like this could be coached in real time using an app or could be flagged for inhaler use training by a health care professional at a later time. In addition, since these systems are only using audio samples, similar results could probably be obtained using a smartwatch with a microphone.

Two other studies observed the movement patterns of patients with Parkinson disease [24,29]. Both of these systems were able to classify the medication status of the patient with an accuracy of 78% or higher, one using body worn sensors and the other using a stationary sensor mounted in a room. This level of accuracy is impressive and has potential that goes beyond simple medication adherence. Systems like this could be used to help inform clinicians of when a patient’s medications need to be adjusted. This is helpful for patients in more advanced stages of degenerative diseases who may have difficulty communicating their current status to care providers. Three systems also classified medication administration using only movement sensors in a smartwatch. These studies were able to achieve a classification accuracy of 78.6%, which is impressive, but this was in a controlled setting with the number of possible activities restricted to a small list [20,21,25]. Determination of medication administration using only movement sensors in a smartphone is an attractive option since it allows tracking with devices that many patients are already using. However, this technology still requires more testing in realistic environments before it can be trusted to accurately determine administration.

### Discussion of Studies That Monitored and Intervened to Improve Adherence

A total of 10 studies also monitored medication adherence and introduced an intervention to improve it [33-42]. Some of the works within this group used many sensors in conjunction with alerts to provide context-aware reminders [33-36]. Two systems using context-aware reminder systems were able to keep patients at an adherence level of 92% or higher [33,34]. This is an impressive level of adherence, but these systems were intrusive and required the use of a large number of sensors throughout the living spaces of the user. Additionally, only 1 of these studies compared its results against a traditional reminder system, and they did so with the results of only a single patient [34]. Furthermore, none of these studies mentioned any effort to establish if users were comfortable with the level of monitoring required to produce a context-aware reminder system. This is potentially an oversight since these systems require the use of multiple sensors throughout a person’s home. The companies creating smart devices would also need to be open to data sharing with their competitors if systems like this are going to be possible in the future. Other works that included medication monitoring with an intervention used conversational AI to communicate with patients [38-40]. All of the conversational systems included reminders and were able to interpret free-text responses provided by patients. Additionally, all of the conversational AI systems were compared against a control group to show if they improved adherence. Two of the three conversational AI systems significantly improved adherence over more traditional reminder systems. One of the conversational AI systems improved refill rates by 14.07% and the other improved average adherence by more than 20% over the study’s duration [39,40]. However, the third conversational AI system did not significantly improve medication adherence when compared to a more traditional system [38]. In general, conversational AI systems are further along in development, allowing the authors to focus more on comparing their systems to more traditional solutions.

### Conclusions

#### Conclusions About Studies That Predicted Medication Adherence

Machine learning also has the potential to substantially improve medication adherence. However, the development of this technology must be well guided to ensure optimal outcomes. The information presented in this review indicates that some predictors remain significant across multiple studies. The creation of more generalizable models that can be quickly adapted to new data sets may make prediction of medication adherence a less time-consuming endeavor in the future. Higher quality measures of adherence status should also be adopted, when possible, to ensure that predictions are based on accurate data. Currently, machine learning has the ability to predict medication adherence with a good level of accuracy. These predictions should be paired with targeted interventions to help prevent medication adherence issues before they occur. However, careful evaluation of models is still paramount to avoid wasting resources on systems that overpredict medication nonadherence. More work also needs to be done to identify predictors of medication adherence in free-text documents. Currently, a lot of medical data is in a free-text format, and this is especially the case for less advanced systems with less structured documentation.

#### Conclusions About Studies That Monitored Medication Adherence

Moreover, systems that monitor medication adherence can accurately classify inhaler use actions, even in the presence of background noise. This technology can be used to track inhaler adherence but can also be taken a step further, allowing for directed interventions aimed at improving inhaler use technique. Adherence monitoring systems are also currently capable of accurately determining the medication status of patients with Parkinson disease using movement data. This information could be used beyond mere adherence by providing clinicians with information that could be used to guide dosage adjustments. This is particularly helpful in a population that is likely to have difficulty communicating their struggles to providers. Systems are also being developed to track medication administration using only smartwatch movement sensors. However, these systems are still in the early development phase and are only being asked to differentiate between a small handful of different activities. These systems need to maintain a good level of classification accuracy in real-world environments before they can offer any clinical utility. However, if these systems are able to achieve this feat they will be highly attractive since they allow for unobtrusive monitoring with common devices that many patients already wear.

#### Conclusions About Studies That Monitored and Intervened to Improve Adherence

Context-aware reminder systems have shown that they can help patients to achieve a high level of adherence but do so in an intrusive fashion. Studies need to be conducted to evaluate the acceptability and desirability of systems like this. These systems should also be compared against traditional reminder systems to make sure they are actually improving adherence. Conversational AI systems aimed at improving medication adherence are already starting to be deployed, and a few have significantly improved adherence over traditional reminder systems. One advantage of these systems is that people can interact with them using the spoken word, so they may be more usable for people who have difficulty interacting with systems requiring the ability to use a computer or smartphone.

### Limitations

The analysis of this topic was limited to five important databases, and some relevant articles from other sources may have been missed. Different grouping strategies might have also added additional insights but were not attempted since they are outside the scope of this paper.

## Appendix

Multimedia Appendix 1 Supplementary material.

## Abbreviations

AI: artificial intelligence
LAIR: Laboratory of Applied Informatics Research
MeSH: Medical Subject Headings
PRISMA-ScR: Preferred Reporting Items for Systematic Reviews and Meta-Analyses Extension for Scoping Reviews
SVM: support vector machine
99DOTS: Directly Observed Treatment Short Course
