# Supplementary material for: Machine Learning and Medication Adherence: Scoping Review
Source: JMIRx Med. 2021 Nov 24;2(4):e26993. doi: 10.2196/26993 (PMC10414315; doi:10.2196/26993)
Supplement: Multimedia Appendix 1 [file xmed_v2i4e26993_app1.pdf]

Appendix A.

Search Queries

PubMed (Probably only include one example in the final draft)

((Artificial Intelligence[Mesh] OR artificial-intelligence[text word] OR “artificial intelligence”[text word] OR Big Data[Mesh] OR “big data”[text word] OR “computational analysis”[text word] OR Data Mining[Mesh] OR “data mining”[text word] OR data-mining[text word] OR deep-learning[text word] OR Deep Learning[Mesh] OR “deep learning”[text word] OR “forecast model”[text word] OR Machine Learning[Mesh] OR “machine learning”[text word] OR machine-learning[text word] OR Natural Language Processing[Mesh] OR “natural language processing”[text word] OR "Neural Networks, Computer"[Mesh] OR “neural network”[text word] OR neural-network[text word] OR “prediction network”[text word] OR “predictive modeling”[text word] OR “time series prediction”[text word]) AND ("Medication Adherence"[Mesh] OR “Medication Adherence”[text word] OR “Medication Nonadherence”[text word] OR “Medication Noncompliance”[text word] OR “Medication Non-Adherence”[text word] OR “Medication Non Adherence”[text word] OR “Medication Persistence”[text word] OR “Medication Compliance”[text word] OR “Medication Non-Compliance”[text word] OR “Medication Non Compliance”[text word]))

Scopus

(('big data'/exp) OR “big data” OR ('artificial intelligence'/exp) OR “artificial intelligence” OR “artificial-intelligence” OR “computational analysis” OR ('data mining'/exp) OR “data mining” OR “data-mining” OR ('deep learning'/exp) OR “deep learning” OR “deep-learning” OR “forecast model” OR ('machine learning'/exp) OR “machine learning” OR “machine-learning” OR ('natural language processing'/exp) OR “natural language processing” OR “nlp” OR ('artificial neural network'/exp) OR “artificial neural network” OR “neural network” OR “prediction network” OR “predictive modeling” OR “time series prediction”) AND (('medication compliance'/exp) OR“medication adherence” OR “medication-adherence” OR “medication nonadherence” OR “medication noncompliance” OR “medication non-adherence” OR “medication noncompliance” OR “medication non-adherence” OR “medication non adherence” OR “medication persistence” OR “medication compliance” OR “medication non-compliance” OR “medication non compliance”)

IEEE

((“artificial intelligence” OR artificial-intelligence OR “big data” OR “computational analysis” OR “data mining” OR data-mining OR deep-learning OR “deep learning”

OR “forecast model” OR “machine learning” OR machine-learning OR “natural language processing” OR “neural network” OR neural-network OR “prediction network” OR “predictive modeling” OR “time series prediction”) AND (“medication adherence” OR “medication nonadherence” OR “medication noncompliance” OR “medication non-adherence” OR “Medication non adherence” OR “medication persistence” OR “medication compliance” OR “medication non-compliance” OR “Medication Non Compliance”))

ACM Digital Library

[[All: "artificial intelligence"] OR [All: artificial-intelligence] OR [All: "big data"] OR [All: "computational analysis"] OR [All: "data mining"] OR [All: data-mining] OR [All: deep-learning] OR [All: "deep learning"] OR [All: "forecast model"] OR [All: "machine learning"] OR [All: machine-learning] OR [All: "natural language processing"] OR [All: "neural network"] OR [All: neural-network] OR [All: "prediction network"] OR [All: "predictive modeling"] OR [All: "time series prediction"]] AND [[All: "medication adherence"] OR [All: "medication nonadherence"] OR [All: "medication noncompliance"] OR [All: "medication non-adherence"] OR [All: "medication non adherence"] OR [All: "medication persistence"] OR [All: "medication compliance"] OR [All: "medication non-compliance"] OR [All: "medication non compliance"]]

Web of Science

((“artificial intelligence” OR artificial-intelligence OR “big data” OR “computational analysis” OR “data mining” OR data-mining OR deep-learning OR “deep learning” OR “forecast model” OR “machine learning” OR machine-learning OR “natural language processing” OR “neural network” OR neural-network OR “prediction network” OR “predictive modeling” OR “time series prediction”) AND (“medication adherence” OR “medication nonadherence” OR “medication noncompliance” OR “medication non-adherence” OR “Medication non adherence” OR “medication persistence” OR “medication compliance” OR “medication non-compliance” OR “Medication Non Compliance”)

Appendix B.

Table 1. Data Charting of Title, Study Goal, Disease State, Date, Publication Type, Database Type

Title	Study Goal	Disease state	Publication Date	Publication type
A machine learning approach for medication adherence monitoring using body-worn sensors	To develop a system that monitors medication adherence using a watch that tracks patient movements.	chronic disease	2016	Conference Proceedings
A Scalable Smartwatch-Based Medication Intake Detection System Using Distributed Machine Learning	To create a system that monitors medication adherence using a smartwatch.	general	2020	Journal Article
A Sensing-Based Framework for Medication Compliance Monitoring	To establish a system that uses sensors within a pill-bottle to monitor medication adherence and patient activity.	general	2019	Conference Proceedings
An artificial neural network classification of prescription non-adherence	To investigate the use of artificial neural networks for classifying reasons for medication non-adherence.	general	2017	Journal Article
An mHealth System for Monitoring Medication Adherence in Obstructive Respiratory Diseases Using Content Based Audio Classification	To develop a system that monitors medication adherence using audio recordings of inhaler use.	asthma or COPD	2018	Journal Article

Application of support vector machine for prediction of medication adherence in heart failure patients	To determine predictors of medication adherence in heart failure patients using SVM.	heart failure	2010	Journal Article
Assessment of medication adherence in respiratory diseases through deep sparse convolutional coding	To create a system that monitors medication adherence using audio recordings of inhaler use.	asthma or COPD	2019	Conference Proceedings
Commercial devices-based system designed to improve the treatment adherence of hypertensive patients	To refine a system that uses a variety of patient monitoring devices to provide context aware medication reminders.	hypertension	2019	Journal Article
Continuous activity monitoring and intelligent contextual prompting to improve medication adherence.	To develop a system that continuously monitors patient activities to provide context aware medication reminders.	general	2007	Journal Article
Determining hypertensive patients' beliefs towards medication and associations with medication adherence using machine learning methods	To use machine learning techniques to predict adherence levels of hypertensive patients.	hypertension	2020	Journal Article

Effect of copayment policies on initial medication non-adherence according to income: A population-based study	To examine the impact of changes in copayment on medication adherence using machine learning methods.	general	2018	Journal Article
Effect of Home Blood Pressure Monitoring via a Smartphone Hypertension Coaching Application or Tracking Application on Adults With Uncontrolled Hypertension: A Randomized Clinical Trial	To determine the effect of an artificial intelligence smartphone coaching app on systolic blood pressure levels.	hypertension	2020	Journal Article
Estimation of Heart Failure Patients Medication Adherence through the Utilization of Saliva and Breath Biomarkers and Data Mining Techniques	To estimate medication adherence of heart failure patients using machine learning techniques that take advantage of saliva and breath biomarkers.	heart failure	2017	Conference Proceedings
Identifying patients with cost-related medication non-adherence: a big-data approach	To identify patients at risk of cost related medication non-adherence (CRN) using big data from survey data and electronic health records.	general	2018	Journal Article

Impact of social determinants of health and demographics on refill requests by Medicare patients using a conversational artificial intelligence text messaging solution: Cross-sectional study	To evaluate the impact of social determinants of health on the effectiveness of a conversation text messaging system that reminds patients to request refills for their medications.	chronic disease	2019	Journal Article
Improving refill adherence in medicare patients with tailored and interactive mobile text messaging: Pilot study	To determine the effectiveness of a conversational text messaging reminder system in patients with chronic diseases and a history of adherence issues.	chronic disease	2018	Journal Article
Initial medication non-adherence: prevalence and predictive factors in a cohort of 1.6 million primary care patients	To determine initial adherence levels to expensive medications that are commonly prescribed and factors that predict it.	expensive medications	2017	Journal Article
Learning to Prescribe Interventions for Tuberculosis Patients Using Digital Adherence Data	To create a system that can predict daily risk of non-adherence, future treatment success, and how best to allocate limited treatment resources.	tuberculosis	2019	Conference Proceedings

Machine learning classification of medication adherence in patients with movement disorders using non-wearable sensors	To determine the feasibility of detecting adherence to Parkinson’s disease medications based on three dimension patient gait data collected using non-wearable sensors.	parkinson's disease	2015	Journal Article
Maximizing the value of mobile health monitoring by avoiding redundant patient reports: Prediction of depression-related symptoms and adherence problems in automated health assessment services	To predict future patient status assessment responses based upon past responses using machine learning methods.	depression	2013	Journal Article
Medhere: A Smartwatch-based Medication Adherence Monitoring System using Machine Learning and Distributed Computing	To refine a system that monitors medication taking behaviors using a smartwatch.	general	2018	Conference Proceedings

Medication adherence prediction through online social forums: A case study of fibromyalgia	To determine if machine learning models trained using data from a previous experiment can accurately predict medication adherence based on data collected from social health forums.	fibromyalgia	2019	Journal Article
Mobi health: A system to improve medication adherence in hypertensive patients	To develop a context aware reminder system that continuously tracks patient activities including medication intake.	hypertension	2018	Conference Proceedings
Monitoring asthma medication adherence through content based audio classification	To determine if the system can accurately monitor medication adherence using audio recordings of inhaler use in real world environments.	asthma or COPD	2016	Conference Proceedings
Monitoring of medication intake using a camera system	To develop a system that uses a camera and an app to monitor medication adherence.	general	2011	Journal Article
Multi-class support vector machines for modeling HIV/AIDS treatment adherence using patient data	To determine how useful SVM is for modeling HIV/AIDS treatment adherence.	HIV	2005	Conference Proceedings

Observing versus predicting: Initial patterns of filling predict long-term adherence more accurately than high-dimensional modeling techniques	To compare the effectiveness of machine learning based approaches against simpler methods for predicting medication adherence.	hyperlipidemia	2016	Journal Article
PatientSense: Patient Discrimination from in-Bottle Sensors Data	To develop a system that can determine a person's identity when they remove pills from a smart pill bottle.	general	2019	Conference Proceedings
PDMove: Towards Passive Medication Adherence Monitoring of Parkinson's Disease Using Smartphone-Based Gait Assessment	To refine a system that uses smartphone sensors to study the gait of patients with Parkinson's disease to determine their medication state.	parkinson's disease	2019	Journal Article
Predicting adherence of patients with HF through machine learning techniques	To predict if heart failure patients will be adherent to medication, nutrition, and exercise regimes using machine learning methods.	heart failure	2016	Journal Article
Predicting medical non-adherence using natural language processing	To develop a system that uses natural language processing to predict treatment non-adherence based on discharge summaries.	general	2017	Conference Proceedings

Prediction of persistence of combined evidence-based cardiovascular medications in patients with acute coronary syndrome after hospital discharge using neural networks	To identify important adherence predictors for patients with acute coronary syndrome using neural networks and logistic regression.	ACS	2011	Journal Article
Predictors of adherence to nicotine replacement therapy: Machine learning evidence that perceived need predicts medication use	To identify and characterize subgroups of smokers based on adherence to nicotine replacement therapy.	smoking cessation	2019	Journal Article
Primary non-adherence to medication and its drivers in Poland: Findings from the electronic prescription pilot analysis	To determine the prevalence and drivers of primary non-adherence in Polish patients with chronic disease.	chronic disease	2019	Journal Article
Recognition of Breathing Activity and Medication Adherence using LSTM Neural Networks	To develop a system that monitors medication intake using audio recordings of inhaler use.	asthma or COPD	2019	Conference Proceedings

Risk for Medication non-adherence Among Medicaid Enrollees With Fibromyalgia: Development of a Validated Risk Prediction Tool	To create and validate a risk assessment tool called the prescription medication Non-adherence Prediction Tool (Rx-NAPT) to predict medication non-adherence in patients with fibromyalgia	fibromyalgia	2019	Journal Article
Spark-based classification algorithms for daily living activities	To establish a system that monitors medication adherence in patients with dementia using body worn sensors.	dementia	2018	Conference Proceedings
Toward Naturalistic Self-Monitoring of Medicine Intake	To refine a context aware medication reminder system that uses a cell phone application and a smart medication bottle.	general	2017	Conference Proceedings
UbMed: A ubiquitous system for monitoring medication adherence	To improve a context aware medication reminder system that gathers data from multiple smart devices.	general	2016	Conference Proceedings

Use of a Novel Artificial Intelligence Platform on Mobile Devices to Assess Dosing Compliance in a Phase 2 Clinical Trial in Subjects With Schizophrenia	Evaluate the use of an application for measuring medication adherence compared with modified directly observed therapy (mDOT) for a phase 2 clinical trial of the drug ABT-126 (nicotinic agonist).	schizophrenia	2017	Journal Article
Using Artificial Intelligence to Reduce the Risk of non-adherence in Patients on Anticoagulation Therapy	To monitor and improve medication adherence with an AI platform and compare the results to that of direct observed therapy.	ischemic stroke	2018	Journal Article
Utilizing convolution neural networks for the acoustic detection of inhaler actuations	To create a system that monitors medication administration using audio recordings of inhaler use.	asthma or COPD	2015	Conference Proceedings
When chatbots meet patients: One-year prospective study of conversations between patients with breast cancer and a chatbot	To evaluate the impact of using a chat bot to improve breast cancer patient care.	breast cancer	2019	Journal Article

Appendix B. Data Charting Table 2

Title	Actions Related to Adherence	Predictors of Medication Adherence	Machine learning algorithms	Number of Participants
An artificial neural network classification of prescription non-adherence	Prediction of medication adherence	Survey results discussing why people are non adherent. Demographic variables- age, marital status, sex, education, primary source of payment, and 25 variables representing- observational learning/ differential association/ differential reinforcement/ medication adherence self efficacy/ outcome expectations/ personal attitudes towards adherence.	Artificial neural networks	474
Application of support vector machine for prediction of medication adherence in heart failure patients	Prediction of medication adherence	11 variables were used to build the models: gender, age, spouse, education, monthly income, heart failure duration, medication frequency, ejection fraction, MMSE-K, medication knowledge, and NYHA functional class.	SVM	76
Determining hypertensive patients' beliefs towards medication and associations with medication adherence using machine learning methods	Prediction of medication adherence	Age, gender, ethnicity, religion, education level, occupational field, monthly income, marital status, duration of medication intake, comorbid conditions, number of meds per day, medication intake aids, counseling for medications, specific necessity, specific concern, general overuse, general harm, and adherence level	Random forest, support vector regression and ANNs (artificial neural networks) were used to predict adherence. SOM was used to make visualizations.	160

Effect of copayment policies on initial medication non-adherence according to income: A population based study	Prediction of medication adherence	Initial medication non-adherence<br>Patient data- sex, age, place of origin, socioeconomic information, comorbidities<br>Family practioner data- sex, age, type- assigned or substitute/resident<br>Center type- resident training center or other	Segmented logistic regression	3,000,000
Identifying patients with cost-related medication non-adherence a big-data approach	Prediction of medication adherence	Age, Chronic conditions- anemia, asthma, arthritis, back problems, depression, digestive problems, ordering medications when receiving social security check, 4 cost related medication non-adherence behaviors	multivariate logistic regression	559
Initial medication non-adherence prevalence and predictive factors in a cohort of 1.6 million primary care patients	Prediction of medication adherence	Patient variables- gender, age, socioeconomic status (5 urban levels and one rural level), nationality<br>Prescription variables<br>Active diseases- allergies, pain, respiratory, blindness, cardiovascular, mental, neurological, diabetes, digestive, thyroid, number of comorbid conditions<br>Pharmacologic class<br>IMNA rates- 1 month after prescription month, 2 months after PM, 3 months after PM<br>Single prescription filled<br>Practitioner variables- gender, age, assigned GP/substitute/resident<br>Center variable- training centre or not a training centre	Multilevel logistic regression	1,599,286 patients, 6953 general practitioners
Medication adherence prediction through online social forums A case study of fibromyalgia	Prediction of medication adherence	Type of medication, years on treatment, daily intake, dosage, age, gender, out of pocket expense, region of residence, medication adherence	random forest	3044 MEPS 357 social health forum

Multi-class support vector machines for modeling HIV/AIDS treatment adherence using patient data	Prediction of medication adherence	CD4 Count, viral load, drug abuse, alcohol abuse, psychiatric diagnosis, missed clinic visits, housing, living with HIV and perceived HIV-related hospitalization	SVM, ANN	33
Observing versus predicting Initial patterns of filling predict long-term adherence more accurately than high-dimensional modeling techniques	Prediction of medication adherence	Investigator specified variables- age, sex, race, prior medications, etc. (35 in total)<br>Socioeconomic from census data- 208 predictor variables, gives insight into income, education, resources of people in a specific geographic location<br>hd-PS selected variables selected by an algorithm<br>Selects variables from claims data<br>Initial statin fill behavior	Logistic regression, boosted logistic regression	77,703
Predicting adherence of patients with HF through machine learning techniques	Prediction of medication adherence	General information- age, gender, caregiver<br>Allergies and medical condition- KILLIP classification, NYHA class, smoking habits, alcoholism, comorbidities<br>Drug information- current medications, dose, frequency, active substances<br>Biological information related to heart failure and clinical examination data	Random forest, random tree, logistic model trees, J48, rotation forest, SVM, radial basis function, bayesian network, naive bayes, multiple layer perceptron, simple classification and regression tree.	90
Predicting medical non-adherence using natural language processing	Prediction of medication adherence	Keywords that occur frequently in the discharge summaries of non-adherent patients.	Word2Vec was used for natural language processing and logistic regression was used to predict medication adherence.	198 unique discharge summaries

Prediction of persistence of combined evidence based cardiovascular medications in patients with acute coronary syndrome after hospital discharge using neural networks	Prediction of medication adherence	ACS characteristics, medications at discharge, cardiovascular risk factors, last labs, comorbidities, concomitant treatments, demographic data	Artificial neural networks, linear regression	2132
Predictors of adherence to nicotine replacement therapy: Machine learning evidence that perceived need predicts medication use	Prediction of medication adherence	Gender, race, marital status, education, household income, age, age of first cigarette, cigarettes per day, FTCD score (tobacco dependence score), number of past quit attempts, adherence outcomes<br>Wisconsin Beliefs Assessment on Smoking and Cessation<br>Wisconsin Smoking Withdrawal Scale<br>Positive and Negative Affect schedule<br>37 item Brief Wisconsin Inventory of Smoking Dependence Motives- affiliative attachment, affective enhancement, automaticity, loss of control, cognitive enhancement, craving, cue exposure, social and environmental goads, taste and sensory properties, tolerance, weight control.	GUIDE (Generalized, Unbiased, Interaction Detection and Estimation) classification tree	623
Primary non-adherence to medication and its drivers in Poland Findings from the electronic	Prediction of medication adherence	Age, sex, date of prescription, trade name, dose, number of packs, prescribed drug, dispensation date, drug details, drug class, special care program, therapeutic area	Logistic regression	Unknown

prescription pilot analysis				
Risk for Medication non-adherence Among Medicaid Enrollees With Fibromyalgia Development of a Validated Risk Prediction Tool	Prediction of medication adherence	Age, gender, race, days supply, medication used (pregabalin(Lyrica), duloxetine (Cymbalta), milnacipran (Savella)), HMO coverage, emergency room visits<br>FMS comorbidity score- headache/migraine, IBS, insomnia, fatigue/tiredness, muscle weakness, thinking or remembering issues, depression, nervousness	Multivariable logistic models	6,626
A machine learning approach for medication adherence monitoring using body-worn sensors	Monitoring of medication adherence	Predictors were created from movement data collected using wrist worn sensors.	decision tree classification algorithm	10
A Scalable Smartwatch Based Medication Intake Detection System Using Distributed Machine Learning	Monitoring of medication adherence	Predictors were generated from movement data collected using a smartwatch.	Random forest, gradient boosted tree, logistic regression, SVM (support vector machine)	24
A Sensing-Based Framework for Medication Compliance Monitoring	Monitoring of medication adherence	Predictors were made using pill bottle movement data and cap status.	binary SVM, multi-class SVM, and random forest	9
Assessment of medication adherence in respiratory diseases through deep	Monitoring of medication adherence	Predictors were developed using audio recordings of inhaler use.	Convolutional neural networks	12

sparse convolutional coding				
Machine learning classification of medication adherence in patients with movement disorders using non-wearable sensors	Monitoring of medication adherence	Predictors developed using data from non-wearable sensors that captured the three dimensional position, velocity and acceleration of the participants' joints.	Naive bayes, IBK, C4.5, J48, SVM, and random forest	7
Medhere A Smartwatch-based Medication Adherence Monitoring System using Machine Learning and Distributed Computing	Monitoring of medication adherence	Predictors generated using data from smartwatch sensors (accelerometer and gyroscope) Movement data from six different activities- medication intake (opening bottle, placing pill in mouth, closing medication bottle) with watch on non dominant wrist, medication intake with watch on dominant wrist, walking, texting, writing with a pen, drinking water	random forest	6
Monitoring asthma medication adherence through content based audio classification	Monitoring of medication adherence	Predictors made using audio recordings of inhaler use.	SVM, random forest, and AdaBoost	5
Monitoring of medication intake using a camera system	Monitoring of medication adherence	Predictors were generated by using a camera system to identify the participant's hands, head, and medication bottle	Petri net	1
PatientSense Patient Discrimination from in-Bottle Sensors Data	Monitoring of medication adherence	Predictors created using accelerometers mounted on the pill bottle body and cap.	Binary SVM, random forest	16

PDMove Towards Passive Medication Adherence Monitoring of Parkinson's Disease Using Smartphone Based Gait Assessment	Monitoring of medication adherence	Predictors developed using gait data collected by cell phone sensors before and after medication administration.	Deep neural network	247
Recognition of Breathing Activity and Medication Adherence using LSTM Neural Networks	Monitoring of medication adherence	Predictors generated using data from audio recordings of inhaler use.	discussed under main study results	3
Spark-based classification algorithms for daily living activities	Monitoring of medication adherence	Predictors made using data from 4 sensors placed on the left ankle, right wrist, chest and right hip	Random forest used for predictor selection. Classification algorithms- logistic regression, decision tree, random forest, and multilayer perceptron	2 data sets The 1st had only 2 subjects The 2nd had 19 subjects
Utilizing convolution neural networks for the acoustic detection of inhaler actuations	Monitoring of medication adherence	Predictors developed using audio recordings of inhaler use.	Convolutional neural network	5
An mHealth System for Monitoring Medication Adherence in Obstructive Respiratory Diseases Using Content Based Audio	Monitoring of medication adherence and an intervention to improve medication adherence	Predictors were created using audio recordings of inhaler use	GMMs, SVM, random forest, and ADABoost	12

Classification				
Commercial devices-based system designed to improve the treatment adherence of hypertensive patients	Monitoring of medication adherence and an intervention to improve medication adherence	Predictors generated using the data collected from commercial devices.	C4.5, J48, RandomTree, and RepTree	Tested on group teammates and a database from another study was used to validate the system.
Continuous activity monitoring and intelligent contextual prompting to improve medication adherence	Monitoring of medication adherence and an intervention to improve medication adherence	Predictors were made using data from stationary smart devices within the home and body worn sensors.	Dynamic bayesian network	11
Effect of Home Blood Pressure Monitoring via a Smartphone Hypertension Coaching Application or Tracking Application on Adults With Uncontrolled Hypertension A Randomized Clinical Trial	Monitoring of medication adherence and an intervention to improve medication adherence	medication adherence- no missed doses vs any missed doses using a 4 day recall, number of antihypertensives used from different drug classes, number of medication increases or substitutions during the study, self confidence using the system / controlling blood pressure / knowing when medications changes were needed / performing non-medication behaviors to control blood pressure, dietary approaches to STOP hypertension adherence, physical activity, sleep duration	Machine learning algorithms used for conversational systems are not mentioned.	297
Improving refill adherence in medicare patients with tailored and interactive mobile text messaging Pilot study	Monitoring of medication adherence and an intervention to improve medication adherence	Patient information- phone number, drug names, gender, name, mobile opt in, level of adherence, date of birth	Machine learning algorithms used for conversational systems are not mentioned.	3 month program 5-14 thousand patients per week

Mobi health A system to improve medication adherence in hypertensive patients	Monitoring of medication adherence and an intervention to improve medication adherence	Predictors were generated using data from home monitoring devices.	J48, RepTree, random forest, Random tree	1100 instances of medication intake
Toward Naturalistic Self-Monitoring of Medicine Intake	Monitoring of medication adherence and an intervention to improve medication adherence	Predictors were created using sensor data from accelerometers.<br>App forms- summary, mood, pain, intake	Random forest, RIPPER, Bayesian Networks, SVM, and Neural Networks	3
UbMed A ubiquitous system for monitoring medication adherence	Monitoring of medication adherence and an intervention to improve medication adherence	Predictors were generated using a variety of different types of sensor data.	J48, Rep tree and Random tree	
Using Artificial Intelligence to Reduce the Risk of non-adherence in Patients on Anticoagulation Therapy	Monitoring of medication adherence and an intervention to improve medication adherence	Predictors created using video recordings of the patient taking the medication.	Specific algorithms not mentioned.	28
When chatbots meet patients One-year prospective study of conversations between patients with breast cancer and a chatbot	Monitoring of medication adherence and an intervention to improve medication adherence	Predictors created from patient responses to chat bot.	Machine learning algorithms used for conversational system not mentioned.	61

Impact of social determinants of health and demographics on refill requests by Medicare patients using a conversational artificial intelligence text messaging solution Cross-sectional study	Prediction of medication adherence, monitoring of medication adherence, and an intervention to improve medication adherence	Age, gender, spoken language, address- used to determine social determinants of health index, race, ethnicity, mobile phone number, opt-in status, refill drugs , responses to messaging system- could be structured (0, 1, 2, 3, 4, 5, 6, 7867, ayuda, help, mail, resub, stop, and stopall) or unstructured	Machine learning algorithms used for conversational systems are not mentioned. Neural network perceptron model used to predict reply likelihood.	99,217
Learning to Prescribe Interventions for Tuberculosis Patients Using Digital Adherence Data	Monitoring of medication adherence and an intervention to improve medication adherence	Time-series predictors- call data indicating if a medication was taken or missed, cumulative total of all missed doses Static predictors- weight band, age band, gender, treatment center ID, other predictors were created from call logs to capture the patient's behavior.	Linear regression, Random forest, SVM, and a deep network named LEAP.	17,000

Use of a Novel Artificial Intelligence Platform on Mobile Devices to Assess Dosing Compliance in a Phase 2 Clinical Trial in Subjects With Schizophrenia	Monitoring of medication adherence and an intervention to improve medication adherence	AiCure uses AI to visually confirm medication administration<br>Uses face recognition and computer vision<br>Identifies the patient the drug and the act of ingestion<br>Flexible with 6 different ways of measuring adherence<br>Visual confirmation, self reported via the app, self reported during a phone call, missed dose, skipped dose, dose taken in clinic<br>Adherence based on number of doses captured by the AI platform relative to the number of planned doses for the week<br>mDOT 22 subject monitored by using this methods, medication taken at a clinic while being observed 3 times weekly<br>Medication taken once daily so not every administration was witnessed<br>Pharmacokinetic blood samples collected at weeks 2,4,6,10, 12,16,18,22,24<br>Adherent if they had measurable drug of interest in their system	see predictors section	53
Estimation of Heart Failure Patients Medication Adherence through the Utilization of Saliva and Breath Biomarkers and Data Mining Techniques	Prediction of medication adherence and monitoring of medication adherence	General Information, allergies, medical conditions, drugs, biological heart failure data, clinical examinations, adherence, biomarkers- uric acid, tumor necrosis factor, cortisol, 8-iso prostaglandin F2a, isoprene, and acetone	Random forest, logistic model trees, J48, classification/regression tree-CART, rotation forest, radial basis function network, SVM, bayesian network, and naive bayes.	29

Maximizing the value of mobile health monitoring by avoiding redundant patient reports: Prediction of depression-related symptoms and adherence problems in automated health assessment services	Monitoring of medication adherence and an intervention to improve medication adherence	Date of patient enrollment, number of complete assessments, sociodemographic data/clinical characteristics collected at baseline, age, gender, education, baseline depression severity, hospital admission in year prior to study, physical functioning as reported by the SF-12, number of comorbid chronic medication conditions<br>Collected during IVR calls- depressive symptom severity, perceived general health status, antidepressant adherence, spending time in bed due to mental issues<br>Patient health questionnaire (PHQ-9) answered over the phone	Multivariate logistic regression	208

Appendix B. Data Charting Table 3

Title	Actions Related to Adherence	Data Collection Method	Adherence Measurement	Limitations
An artificial neural network classification of prescription non-adherence	Prediction of medication adherence	Survey	Survey used to determine reasons for non-adherence.	This system needs to be incorporated into the EHR or clinicians will not use it. This technology needs to be accompanied with an actual intervention to improve medication adherence. This technology could be used to guide which interventions may work best for a specific patient. Response bias also existed since many people that sent responses had too much missing data to be used. Also, a lot of people did not respond but that is typical.<br>Would require additional data collection in real world application which is problematic given how much information patients are already expected to provide.
Application of support vector machine for prediction of medication adherence in heart failure patients	Prediction of medication adherence	Self-reported questionnaire	Self reported adherence using a questionnaire	Small sample size. Results are based on self reported adherence data.
Determining hypertensive patients' beliefs towards medication and associations with medication adherence using machine learning methods	Prediction of medication adherence	Questionnaires	Self reported adherence using a questionnaire	Small number of participants. Validation of models is difficult with machine learning due to the complexity of the computational process. SOM used to create visualization to help deal with this issue. The system needs to go through a more stringent validation process before it can be deployed in a clinical setting.

Effect of copayment policies on initial medication non-adherence according to income: A population based study	Prediction of medication adherence	Dispensing and invoicing data	Not filling a prescription within a specific period of time from when it was prescribed.	The number of data points were lower than recommended for a segmented regression analysis reducing the available sample size. The sample size may have been inadequate to measure the true impact of the system in certain populations (most problematic for the high income population). Dispensing a medication does not prove it was ingested. The data also had missing values which were simulated using imputation. Computational cost required the use of simple imputation methods over more advanced techniques. Since the two copayment policies occurred simultaneously the impact of each one individually could not be determined.
Identifying patients with cost-related medication non-adherence a big-data approach	Prediction of medication adherence	Data from surveys and EHR data	Self reported cost related non-adherence	Differences in coverage beyond medicare were not considered. Patients may be selectively nonadherent with meds they do not consider as important. Small sample size, only one year of data, and conducted at a single location.
Initial medication non-adherence prevalence and predictive factors in a cohort of 1.6 million primary care patients	Prediction of medication adherence	Catalan PC system in Spain provided prescription and invoicing data.	Medication considered not initiated if not picked up within the month after it was originally prescribed.	Patient beliefs and feelings (beliefs about medications, illness, adverse effects past/present, relationship with PCP) not accounted for in this study. Also, the study did not directly measure medication administration.

Medication adherence prediction through online social forums A case study of fibromyalgia	Prediction of medication adherence	Survey data- medical expenditure panel survey (MEPS)<br>MEPS used MRA(medication refill-adherence) to determine adherence.<br>Social health forum data- PatientsLikeMe<br>Self reported in one of four categories of adherence	MEPS used medication refill data to determine adherence.<br>PatientsLikeMe used self reported adherence.	Patients may not be similar enough to those from the original dataset. Some of the predictors did not transfer well. Adherence reported differently in the two data sources making generation of consistent results difficult. Low prediction accuracy. Adherence is not measured directly by either study.
Multi-class support vector machines for modeling HIV/AIDS treatment adherence using patient data	Prediction of medication adherence	Qualitative patient interview, medical chart review, and provider interview	Self reported adherence	Small sample size
Observing versus predicting Initial patterns of filling predict long-term adherence more accurately than high-dimensional modeling techniques	Prediction of medication adherence	Medical/pharmacy claims and linked census data	This study measured adherence by determining the proportion of days covered for each patient using pharmacy claims data.	All patients had stable health coverage which is not necessarily realistic. Older study population which can impact adherence rates. Some patients may fill the medication and not take it.
Predicting adherence of patients with HF through machine	Prediction of medication adherence	Data collected from retrospective sources provided by a	Adherence based on clinician estimation	Study did not account for socio-economic status, the patients healthcare system, and patient medication knowledge.

learning techniques		university hospital.		
Predicting medical non-adherence using natural language processing	Prediction of medication adherence	Data was collected from the MIMIC database.	A human annotator manually classified each discharge summary.	The system could be expanded to investigate adherence using a larger variety of clinical notes (nursing notes, social work notes, etc.) The system only looks for single words and can be expanded to also examine combinations of words and phrases.
Prediction of persistence of combined evidence based cardiovascular medications in patients with acute coronary syndrome after hospital discharge using neural networks	Prediction of medication adherence	PREVENIR cross sectional survey	Self-reported adherence	Can be difficult to determine if what the neural network is doing is actually valid due to the complexity of the computation process. Variable reduction methods were used to reduce the complexity of predictions and to help avoid overfitting. Predictions based on self reported adherence.
Predictors of adherence to nicotine replacement therapy: Machine learning evidence that perceived need predicts medication use	Prediction of medication adherence	Baseline data collection and interviews after 4 and 16 weeks	Self reported during phone interviews at 4 and 16 weeks after initiation of NRT.	Self reported adherence is not as reliable as other methods. Adding text messaging reminders to the system may help to improve adherence.

Primary non-adherence to medication and its drivers in Poland Findings from the electronic prescription pilot analysis	Prediction of medication adherence	All e-prescriptions issued in Poland in 2018.<br>47 key drugs of importance were focused upon due to their importance in treating common chronic conditions.	Not filling a prescription within 1 month from the date of issuance.	Does not provide insight into why a specific prescription was not filled. Not possible to identify individual patients with the data provided. Could not determine the number of patients that were initially non-adherent. Could not determine the rate of primary non adherence to different drugs prescribed to the same patient. Also, electronic prescriptions are not available everywhere in Poland so there is some selection bias based on location.
Risk for Medication non-adherence Among Medicaid Enrollees With Fibromyalgia Development of a Validated Risk Prediction Tool	Prediction of medication adherence	Data provided by South Carolina medicaid	Medication claims data<br>MPR (medication possession ratio)	Probably overestimates adherence since claims data does not prove the medication was ingested. Only Five predictors from claims data included in the model, other predictors (medication preference, socioeconomic status, using multiple classes of medications) may also be important. May not be generalizable to other patient populations
A machine learning approach for medication adherence monitoring using body-worn sensors	Monitoring of medication adherence	Data collected using wrist worn sensors.	Medication taking was integrated into the experimental process.	The system needs to be tested in real world conditions where the patient can do a large variety of different activities.
A Scalable Smartwatch Based Medication Intake Detection System Using Distributed Machine Learning	Monitoring of medication adherence	Data collected using wrist worn sensors.	Medication taking was integrated into the experimental process.	Additional sensors could be added to the watch in the future potentially increasing the F1 score of the system even more.

A Sensing-Based Framework for Medication Compliance Monitoring	Monitoring of medication adherence	Data collected using medication bottles equipped with sensors.	Video recording used to establish ground truth.	Increasing the number of sensors may help the system to determine medication taking behaviors with a higher degree of accuracy.
Assessment of medication adherence in respiratory diseases through deep sparse convolutional coding	Monitoring of medication adherence	Audio recordings of inhaler use	Medication taking was integrated into the experimental process.	System requires further testing in real world settings.
Machine learning classification of medication adherence in patients with movement disorders using non-wearable sensors	Monitoring of medication adherence	Non-wearable sensors (microsoft kinect sensors) used to determine the movement of the patient Patients monitored first off of their medication and then on their normal medication routine	Medication status was integrated into the experimental process.	Requires additional testing in a more realistic environment. The off drug state may not be similar enough to a non-adherent state. Does not account for change in gait due to disease progression. Many confounding variables that can affect gait. Small sample size. Only designed to determine feasibility of using this type of system.
Medhere A Smartwatch-based Medication Adherence Monitoring System using Machine Learning and Distributed Computing	Monitoring of medication adherence	Smart watch collected information using: inertial sensors, accelerometer, gyroscope to monitor a series of actions done when a patient takes their medication	Medication taking was integrated into the experimental process.	Only 6 participants and one site. Still in the early stages of development. May not work well for patients that have physical limitations or movement disorders.

Monitoring asthma medication adherence through content based audio classification	Monitoring of medication adherence	Audio recording device attached to metered dose inhaler	Medication taking was integrated into the experimental process.	Currently only evaluating if specific medication taking steps are occurring instead of evaluating the entire process as a whole.
Monitoring of medication intake using a camera system	Monitoring of medication adherence	Video recording	Medication taking was integrated into the experimental process.	Technology is still in development. Not advanced enough to tell if a person is just interacting with the bottle or taking the medication. Requires additional testing in a more realistic environment. Sensitivity to persons clothes color, skin coverage, items on the table, color of table, color of medication vial, angle of person's head, camera angle, and watches or other items that cover the skin. Only tested on a single actor.
PatientSense Patient Discrimination from in-Bottle Sensors Data	Monitoring of medication adherence	Accelerometer data	Medication taking was integrated into the experimental process.	More fine tuning of the model is required to reduce the number of false positives.
PDMove Towards Passive Medication Adherence Monitoring of Parkinson’s Disease Using Smartphone Based Gait Assessment	Monitoring of medication adherence	Gait sensors in cell phone Accelerometer and gyroscope	Medication status was integrated into the experimental process.	Requires further testing in more realistic environments. The system does not work as well for patients that have a very subtle difference in gait after medication administration.
Recognition of Breathing Activity and Medication Adherence using LSTM Neural Networks	Monitoring of medication adherence	Audio recordings of inhaler use	Audio samples were manually annotated	Requires larger validation studies in a variety of acoustic environments.

Spark-based classification algorithms for daily living activities	Monitoring of medication adherence	Data collected using four sensors placed on the left ankle, right wrist, chest, and right hip.	Taking medication was one of the activities that was done during the data collection process.	System still in the early stages of development and only tested on a small number of participants.
Utilizing convolution neural networks for the acoustic detection of inhaler actuations	Monitoring of medication adherence	Acoustic sensors on MDIs, participants were asked to record inhaler use and environmental sounds	Audio samples were manually annotated	Requires further testing in more realistic conditions.
An mHealth System for Monitoring Medication Adherence in Obstructive Respiratory Diseases Using Content Based Audio Classification	Monitoring of medication adherence and an intervention to improve medication adherence	Audio recording device attached to inhaler	Audio samples manually annotated	The usefulness of the predictors being provided need to be evaluated to ensure that they are adequately meeting patient needs.
Commercial devices-based system designed to improve the treatment adherence of hypertensive patients	Monitoring of medication adherence and an intervention to improve medication adherence	Sensor data- cameras, presence sensors, luminosity sensors, thermometers, smart TVs, smart phones, smart watches, door sensors, blood pressure sensor Medicine cabinet- sensors determine when compartment opened, also uses RFID, and	Medication taking was integrated into the experimental process.	System is still being developed and needs more refinement before larger implementation.

		facial recognition		
Continuous activity monitoring and intelligent contextual prompting to improve medication adherence	Monitoring of medication adherence and an intervention to improve medication adherence	The device collected information on participant activity using motion sensors within their house that communicated to a laptop computer. Also had sensors on the refrigerator, phones, a watch worn by the user, beds, and the front door. Adherence information was collected by using MedTracker which is a pill tray that can detect when it is opened.	Adherence was determined as taking the medication within a 3 hour window of the selected time (90 minutes before or after the time selected by the participant before the study began)	This type of system will fail if the person receiving the alerts is not paying attention to the system or is not home to receive the reminder. Also, this system uses a large number of sensors which may seem invasive.
Effect of Home Blood Pressure Monitoring via a Smartphone Hypertension Coaching Application or Tracking Application on Adults With Uncontrolled Hypertension A Randomized Clinical Trial	Monitoring of medication adherence and an intervention to improve medication adherence	In person assessments were conducted at baseline and at 6 months. Measured blood pressure using a standard automated device. Also measured weight. Survey used to collect the remaining predictors.	Adherence was self reported in this study.	Study may have been too small to measure the impact of the intervention. Intervention group had lower blood pressure but the difference was not significant. The small sample size also did not allow for a robust subgroup analysis. Non-blinded study may have introduced bias but was unavoidable due to the nature of the intervention. Coaching may make users more likely to report socially acceptable answers. Results based on self reported adherence data. Participants were not selected based on how likely they are to use the app. Selecting patients more likely to

				use the app may have improved the results.
Improving refill adherence in medicare patients with tailored and interactive mobile text messaging Pilot study	Monitoring of medication adherence and an intervention to improve medication adherence	Data was collected by Kaiser and through the reminder system.	Adherence was determined by the rate of refill requests.	Did not consider social determinants of health but this was addressed in a future study. System requires further refinement to improve its NLP algorithms. The system only categorizes messages instead of acting on them autonomously.
Mobi health A system to improve medication adherence in hypertensive patients	Monitoring of medication adherence and an intervention to improve medication adherence	Data collected multiple sensors in: computers, tablets, smart TVs, smartphones, smartwatches, medicine cabinets, etc.	Medication taking was integrated into the experimental process.	Still in the early stages of development.
Toward Naturalistic Self-Monitoring of Medicine Intake	Monitoring of medication adherence and an intervention to improve medication adherence	Accelerometer tags on medication bottles and a smartphone app	Medication adherence confirmed by patients using a cell phone application.	The system still requires more fine tuning to reduce the number of false positives.
UbMed A ubiquitous system for monitoring medication adherence	Monitoring of medication adherence and an intervention to improve medication adherence	Data collected using multiple sensors: motion sensors, lighting sensors, cameras, smartphone sensors, smart tvs, and an intelligent medicine cabinet. The cabinet includes an RFID scanner, cameras, lamps, and a speaker.	Medication taking was integrated into the experimental process.	The system needs to be improved in terms of cost of hardware, size of devices, and medication administration detection accuracy.

Using Artificial Intelligence to Reduce the Risk of non-adherence in Patients on Anticoagulation Therapy	Monitoring of medication adherence and an intervention to improve medication adherence	AI platform (AICure), pill count, direct measurement of plasma levels of drug	Pill counts<br>Plasma monitoring<br>AI platform	Some of the authors work for the company that made the product being evaluated.
When chatbots meet patients One-year prospective study of conversations between patients with breast cancer and a chatbot	Monitoring of medication adherence and an intervention to improve medication adherence	Patient responses to chat bot	Self-reported adherence	Some of the authors work for the company that makes the product being evaluated.
Impact of social determinants of health and demographics on refill requests by Medicare patients using a conversational artificial intelligence text messaging solution Cross-sectional study	Prediction of medication adherence, monitoring of medication adherence, and an intervention to improve medication adherence	Responses to messaging system and patient data provided by the healthcare provider. Specifics on collecting patient data were not discussed in detail.	Medication adherence using the interactive messaging system was tracked by looking at refill requests processed using the system.	The system requires further improvement to reduce the number of misinterpreted responses. Data was only collected for three months for this experiment. Patients are not random since they had to opt into digital engagement.
Learning to Prescribe Interventions for Tuberculosis Patients Using Digital Adherence Data	Monitoring of medication adherence and an intervention to improve medication adherence	Data was collected as part of the 99DOTS program.	Self-reported adherence	Data loss due to multiple patients using the same phone number and incomplete records.

Use of a Novel Artificial Intelligence Platform on Mobile Devices to Assess Dosing Compliance in a Phase 2 Clinical Trial in Subjects With Schizophrenia	Monitoring of medication adherence and an intervention to improve medication adherence	Returned blister packs used to track adherence for the clinical trial<br>AI app reported adherence was not used as part of the clinical trial<br>Drug levels measured directly	Blood samples were used to measure the level of medication in the body.	Some of the authors work for the company that made the device being tested. Lack of randomization since patients were allowed to choose between mDOT and the AI application. Small sample sizes in both groups. Plasma concentration collected without regard for the timing of the last dose of medication. However, adherence was considered intact at a low level of drug so this was likely not an issue
Estimation of Heart Failure Patients Medication Adherence through the Utilization of Saliva and Breath Biomarkers and Data Mining Techniques	Prediction of medication adherence and monitoring of medication adherence	Data was collected during the patient's hospitalization.	Clinician assigned according to patient status	Clinician assigned status may not accurately reflect adherence status.
Maximizing the value of mobile health monitoring by avoiding redundant patient reports: Prediction of depression-related symptoms and adherence problems in automated health assessment services	Monitoring of medication adherence and an intervention to improve medication adherence	IVR assessments	Self-reported adherence	Self reported adherence may not be accurate. Focused on several main outcomes including adherence. Increasing the number of assessment intervals should be investigated in future studies.
